# Evaluating the Rate and Causes of Non-candidacy After Laser-Assisted in Situ Keratomileusis (LASIK) Screening

**DOI:** 10.7759/cureus.86618

**Published:** 2025-06-23

**Authors:** Muhammed Jaafar, Kenneth D Han, Mina M Sitto, Preston B Willey, Walker C Kay, Nathan Olson, Kayvon A Moin, Michael T Christensen, Phillip Hoopes, Majid Moshirfar

**Affiliations:** 1 Ophthalmology, University of Arizona College of Medicine - Phoenix, Phoenix, USA; 2 Ophthalmology, Hoopes Vision Research Center, Draper, USA; 3 Ophthalmology, Wayne State University School of Medicine, Detroit, USA; 4 Ophthalmology, Spencer Fox Eccles School of Medicine, Salt Lake City, USA; 5 Ophthalmology, Noorda College of Osteopathic Medicine, Provo, USA; 6 Ophthalmology, Rocky Vista University College of Osteopathic Medicine, Ivins, USA; 7 Ophthalmology, Nassau University Medical Center, East Meadow, USA; 8 Ophthalmology, Wake Forest School of Medicine, Winston-Salem, USA; 9 Ophthalmology, University of Minnesota School of Medicine, Minneapolis, USA; 10 Ophthalmology, Hoopes Vision, Draper, USA; 11 Ophthalmology, Cornea and Refractive Surgery, University of Utah School of Medicine, Salt Lake City, USA; 12 Eye Banking and Corneal Transplantation, Utah Lions Eye Bank, Murray, USA; 13 Ophthalmology, Cornea and Refractive Surgery, Hoopes Vision, Draper, USA

**Keywords:** corneal refractive surgery, emmetropia, hyperopia, keratoconus, lasik, myopia, ophthalmology, presbyopia, rejection, small incision lenticular extraction (smile)

## Abstract

Purpose

To provide an updated characterization of the reasons for laser-assisted in situ keratomileusis (LASIK) non-candidacy.

Methods

A retrospective chart review was conducted on 648 patients (648 right eyes) who presented for LASIK evaluation at a refractive surgery center in Draper, UT, between November 2022 and April 2023. Age, spherical equivalent (SEQ), and keratometry measurements were compared between LASIK candidates and non-candidates. The overall rate of non-candidacy and reasons for exclusion were documented. Subgroup analyses were performed based on age groups, stratifying patients into two cohorts: patients younger than 45 years and those 45 years or older.

Results

The overall LASIK non-candidacy rate was 69.4%, primarily due to emmetropic presbyopia (36.9%), abnormal topography (31.3%), and hyperopic presbyopia (25.6%). Among patients < 45 years, non-candidacy was often due to abnormal topography (43.5%), corneal thinning (24.5%), and severe myopia (20.7%). Compared to LASIK candidates < 45 years, non-candidates had thinner corneas (548.8 ± 29.0 vs 526.4 ± 33.6 µm; *P* < 0.001), and steeper corneas, including K1 (42.7 ± 1.6 vs 43.3 ± 1.5 D; *P* = 0.002) and K2 (43.9 ± 1.6 vs 44.7 ± 1.7 D; *P* < 0.001). In contrast, for patients ≥ 45, presbyopia (emmetropia: 74.2% and hyperopia: 51.6%) was the leading barrier to qualifying for LASIK. Non-candidates in this cohort showed higher mean age (*P* = 0.02) and had less myopia (−0.71 ± 3.03 vs −3.40 ± 2.12 D; *P* < 0.001) compared to their eligible counterparts.

Conclusion

LASIK remains one of the most thoroughly evaluated refractive procedures, with a proven track record of long-term safety, efficacy, and high patient satisfaction. LASIK non-candidacy was found to be influenced by demographics, with distinct corneal patterns across each subgroup. Patients younger than 45 years were primarily excluded due to abnormal topography, corneal thinning, and severe myopia, while patients 45 and older were more often excluded due to presbyopia, specifically emmetropia and hyperopia. The high non-candidacy rate not only reflects the rigorous screening criteria but also the increased sensitivity of diagnostic devices and the expanding surgical alternatives, such as intraocular lenses with advanced optics for multifocal correction. This allows more patients to achieve greater visual satisfaction than was possible two to three decades ago.

## Introduction

Since the first laser-assisted in situ keratomileusis (LASIK) surgery in 1988, it has proven to be a safe and effective procedure for the treatment of mild to moderate myopia, astigmatism, and hyperopia. It is well-regarded for improving both quality of life and work performance and has become the most commonly performed laser eye treatment [1]. As a result of its popularity, more than 7,000 scientific articles have been published regarding LASIK since its inception. Even liposuction, a commonly performed bariatric procedure, has been less scrutinized than LASIK, with a little over 6,000 publications since the 1980s. Furthermore, the long-established safety of LASIK is supported by the U.S. Food and Drug Administration (FDA) approval of over 30 LASIK platforms since its invention in 1988 [2-6]. For comparison, small incision lenticular extraction (SMILE), a procedure for myopia with and without astigmatism, was not approved by the FDA until 2016 [2]. All in all, LASIK surgery has undergone extreme scrutiny and has emerged as an undeniably safe and effective procedure.

However, alternative surgical options for vision correction have been increasing in number, and modern refractive surgeons have more options in their armamentarium than what was available 20-30 years ago. For instance, phakic intraocular lenses (IOLs), such as implantable collamer lens (ICL), are a viable option for high myopes [7,8]. Other options include refractive lens exchange (RLE) for select patients with hyperopia, and newer IOLs with optics that provide extended depth of focus and binocularity at both distance and near. These options are especially beneficial for patients experiencing loss of accommodation, including emmetropic and hyperopic presbyopes [8]. Additionally, SMILE competes with LASIK by offering a similar range of refractive correction and therefore draws from the same candidate pool [9]. For these reasons, the availability of a wider range of refractive procedures allows clinicians the flexibility to select the most appropriate option based on individual patient characteristics and preferences, not due to any shortcomings of LASIK itself, but because more options now exist to meet the diverse needs of patients.

Notably, the prevalence of hyperopia relative to myopia is higher among individuals between the ages of 45 and 65 [10]. With the growing size and longevity of this population, many young baby boomers and Generation X individuals are not content with monovision, instead hoping for binocularity and the ability to re-experience their vision quality as in their 20s and 30s. However, older populations require special considerations, and while LASIK remains the benchmark of refractive surgery, alternative refractive procedures may be more suitable for these populations [11].

Over the past two decades, previous studies have analyzed the main reasons for ineligibility of LASIK surgery in Saudi, Indian, Yemeni, and Midwestern United States (U.S.) cohorts, with the Yemeni and Saudi cohorts showing a candidacy rate of 74.6% and 75.1%, respectively [12-15]. However, since the publication of these studies, the landscape of refractive surgery, the quality of diagnostic devices, and the criteria for candidacy have continued to evolve [2]. This study aims to provide an updated characterization of both the overall LASIK candidacy rate and the rates of various reasons for LASIK non-candidacy for all patients presenting for LASIK consultation at a single refractive surgical center in Draper, UT.

## Materials and methods

This is a retrospective chart review of 648 patients (one right eye per patient to avoid inter-eye bias) who presented for LASIK evaluation at a single refractive surgery center in Draper, UT, over six months (November 2022 to April 2023). The study was approved by the Biomedical Research Alliance of New York Institutional Review Board (IRB) # A20-12-547-823, adhered to the tenets of the Declaration of Helsinki, and HIPAA regulations were followed. The study was also approved by the Hoopes Vision Ethics Committee.

Demographic information was collected for each patient, including age, sex, ocular history, other medical history, and current medications. As part of the screening process, Scheimpflug Pentacam (OCULUS Optikgeräte GmbH, Wetzlar, Germany) from the most recent LASIK evaluation was reviewed to obtain the flat (K1) and steep (K2) meridians of the anterior corneal surface, central corneal thickness, thinnest pachymetry, as well as results from manifest refraction, cycloplegic refraction, and dilated fundus examination. Additionally, NIDEK (San Jose, CA) Placido image topography and the Avanti Widefield optical coherence tomography (OCT) system (Optovue, Fremont, CA) were utilized for epithelial map analysis in each patient. Patients wearing soft contact lenses were instructed to discontinue use for 1 week, those with extended soft contact lenses and soft toric lenses for two weeks, and those with rigid gas permeable contact lenses for six weeks. Reasons for LASIK non-candidacy were recorded from the physician's notes, and topography was reviewed by an ophthalmologist. Importantly, the factors used to determine LASIK candidacy or non-candidacy were based on the individual practice preferences and clinical expertise of physicians at a single institution.

Patients were divided into two groups: LASIK candidates and LASIK non-candidates. The LASIK non-candidate group was divided into several subcategories depending on the reason for non-candidacy as follows: emmetropic presbyopia, hyperopic presbyopia, abnormal topography, corneal thinning (i.e. central corneal thickness [CCT] < 500 µm), severe myopia, keratoconus, unstable refraction, severe hyperopia, steep corneas, other ocular conditions (i.e. amblyopia, Fuch’s dystrophy [16], glaucoma), cataracts, corneal scarring, contraindicated systemic medications (i.e. isotretinoin [17], spironolactone [18], biologics [19]), medical conditions (i.e. uncontrolled autoimmune disorders, collagen vascular diseases, diabetes, etc), and minimal refractive error (Table 1). Some patients who had multiple reasons for LASIK non-candidacy were included in all applicable categories. The frequency of reasons for LASIK non-candidacy was recorded. Patients were stratified by age into two groups: those < 45 years and those ≥ 45 years, which is consistent with the onset of presbyopia. The frequency of LASIK rejection was then analyzed within each group. The top three reasons for non-candidacy in each age group were compared using the chi-square test. Additionally, patients aged 20-30 years, a typical age range for corneal refractive surgery, were stratified to analyze the overall rejection rate within the younger cohort.

**Table 1:** LASIK Non-Candidacy Subcategory Explanations LASIK: laser assisted in situ keratomileusis; UDVA: uncorrected distance visual acuity; ART: Ambrosio Relational Thickness; IHD: index of height decentration; KI: keratoconus index; ISV: index of surface variance; IVA; index of vertical asymmetry; K_m: _mean keratometry; CDVA: corrected distance visual acuity; KCN: keratoconus; SEQ: spherical equivalent.

Subcategory	Explanation
Emmetropic Presbyopia	Patients with no refractive error with UDVA of 20/20 but symptoms of presbyopia requiring reading glasses
Hyperopic Presbyopia	Patients with hyperopic refractive error who have symptoms of presbyopia requiring reading glasses
Unstable Refraction	Any patient whose refractive error within 1 year has changed by > 0.50 D
Abnormal Topographic Indices	Inferior or superior corneal steepening, irregular astigmatism, and Pentacam parameters such as ART Max, progression index, anterior elevation, posterior elevation, Belin/Ambrósio enhanced ectasia display deviation value, IHD, KI, ISV, IVA, and Kmax
Corneal Scarring	Significant corneal scarring from contact lens wear or prior infection involving the anterior one-third of the corneal thickness, with sufficient density to compromise femtosecond laser flap creation
Steep Corneas	Corneas were too steep to reasonably undergo refractive surgery without potential complications
	K_m_ > 47.2 D
	K1 and K2 both > 47 D
	Generalized steep cornea with (both flat and steep meridian) with no sign of KCN or other corneal abnormal topography, but their curvature is > 48 D
Corneal Thinning	Thinnest pachymetry < 500 μm
	Residual stromal bed of < 300 μm after refractive correction
Keratoconus	Patients with KCN on examination by topography or tomography
Cataracts	Patients with cataracts
Other Ocular Condition(s)	Amblyopia: if CDVA < 20/200
	Fuch’s dystrophy [16]
	Glaucoma
Severe Myopia	Sphere > −8.0 D
	Sphere < −8.0D with concurrent corneal thinning (but not meeting the threshold for corneal thinning category)
	Residual stromal bed < 300 µm
Severe Hyperopia	Sphere > +4.0 D
Contraindicated Medications	Isotretinoin [17]
	Systemic spironolactone [18]
	Biologics [19]
Medical Condition	Uncontrolled autoimmune disorders
	Uncontrolled collagen vascular diseases
	Uncontrolled diabetes
	Cancer (not in remission)
Minimal Refractive Error	Patients who did not have sufficient refractive error to justify surgery
	UDVA of 20/20 but have a residual manifest refraction of SEQ ± 0.5 D

Statistical analysis

Microsoft Excel (Microsoft Corporation, 2018), IBM SPSS (version 29.0; SPSS Inc.), and G*Power (version 3.1, Düsseldorf, Germany) were used for all data collection and statistical analysis. To avoid confounding inter-eye variability, only the right eye of each patient was included in statistical analyses. The Kolmogorov-Smirnov test for normality was performed, and the data were found to be normally distributed. Mean age, spherical equivalent (SEQ), and keratometry measurements (K1, K2, and thinnest pachymetry) were compared between the LASIK candidate and LASIK non-candidate groups using analysis of variance (ANOVA) with Tukey’s post hoc test. Chi-square was performed to assess the top three reasons for LASIK non-candidacy between patients < 45 and patients ≥ 45 years old. The effect size was calculated using Cohen's d. G*Power software determined that a sample size of 596 eyes was needed to achieve a statistical power of 0.80, given a P-value of 0.05 and a conservative effect size of 0.25. Tukey’s post hoc test with a sample size of 648 eyes confirmed an overall statistical power of greater than 0.83.

## Results

Out of the 648 total patients, 198 patients were LASIK candidates and 450 were not, resulting in an overall rejection rate of 69.4%. LASIK non-candidacy was more common in patients ≥ 45 (84%, n = 213) than in those < 45 years (60%, n = 237). For patients aged 20-30 years (n = 110), the non-candidacy rate remained unchanged at 60% (n = 66). For all patients in the study, the LASIK non-candidate group was older (43 ± 11 vs 36 ± 9; P < 0.001), had less myopic SEQ (−1.86 ± 3.61 vs −3.48 ± 2.24 D; P < 0.001), higher K1 (43.3 ± 1.4 vs 42.9 ± 1.6 D; P = 0.045) and lower pachymetry values (531.9 ± 33.2 vs 547.6 ± 28.3 µm; P < 0.001) than the LASIK candidate group (Table 2). When stratified by age, the LASIK non-candidates under 45 had higher K1 (43.3 ± 1.5 vs 42.7 ± 1.6 D; P = 0.002) and K2 (44.7 ± 1.7 vs 43.9 ± 1.6 D; P < 0.001), as well as lower pachymetry values (526.4 ± 33.6 vs 548.8 ± 29.0 µm; P < 0.001), compared to LASIK candidates of the same age group. LASIK non-candidate patients ≥ 45 had a higher mean age (52 ± 4 vs 49 ± 4 years; P = 0.02) and less myopic SEQ (−0.71 ± 3.03 vs −3.40 ± 2.12 D; P < 0.001) than the LASIK candidate cohort of the same age.

**Table 2:** Comparison of Demographics, Keratometry, and Pachymetry Between LASIK Candidate and Non-Candidate Cohorts *Statistically significant difference between candidate and non-candidate groups per patient cohort using analysis of variance (ANOVA) (P < 0.05). Values expressed as mean ± standard deviation. LASIK: laser-assisted in situ keratomileusis; SEQ: spherical equivalent.

	LASIK Candidate	LASIK Non-Candidate	P	F
All Patients (n = 648)	n = 198	n = 450		
Female/Male N (%)	72/126 (36/64)	227/223 (50/50)		
Age (years)	36 ± 9	43 ± 11	< 0.001*	32.63
SEQ (D)	−3.48 ± 2.24	−1.86 ± 3.61	< 0.001*	41.18
K1 (D)	42.9 ± 1.6	43.3 ± 1.4	0.045*	2.57
K2 (D)	44.1 ± 1.5	44.5 ± 1.6	0.142	4.33
Thinnest Pachymetry (µm)	547.6 ± 28.3	531.9 ± 33.2	< 0.001*	17.28
< 45 years old (n = 395)	n = 158	n = 237		
Female/Male N (%)	61/97 (39/61)	151/86 (64/36)		
Age (years)	33 ± 6	33 ± 7	0.902	3.43
SEQ (D)	−3.33 ± 2.28	−3.57 ± 3.35	0.851	15.08
K1 (D)	42.7 ± 1.6	43.3 ± 1.5	0.002*	2.32
K2 (D)	43.9 ± 1.6	44.7 ± 1.7	< 0.001*	3.01
Thinnest Pachymetry (µm)	548.8 ± 29.0	526.4 ± 33.6	< 0.001*	13.04
≥ 45 years old (n = 253)	n = 40	n = 213		
Female/Male N (%)	11/29 (28/73)	76/137 (36/64)		
Age (years)	49 ± 4	52 ± 4	0.020*	3.50
SEQ (D)	−3.40 ± 2.12	−0.71 ± 3.03	< 0.001*	22.68
K1 (D)	43.5 ± 1.6	43.4 ± 1.4	0.978	1.3
K2 (D)	44.5 ± 1.6	44.3 ± 1.6	0.893	2.22
Thinnest Pachymetry (µm)	548.8 ± 29.0	540.0 ± 31.0	0.311	5.44

**Figure 1:**
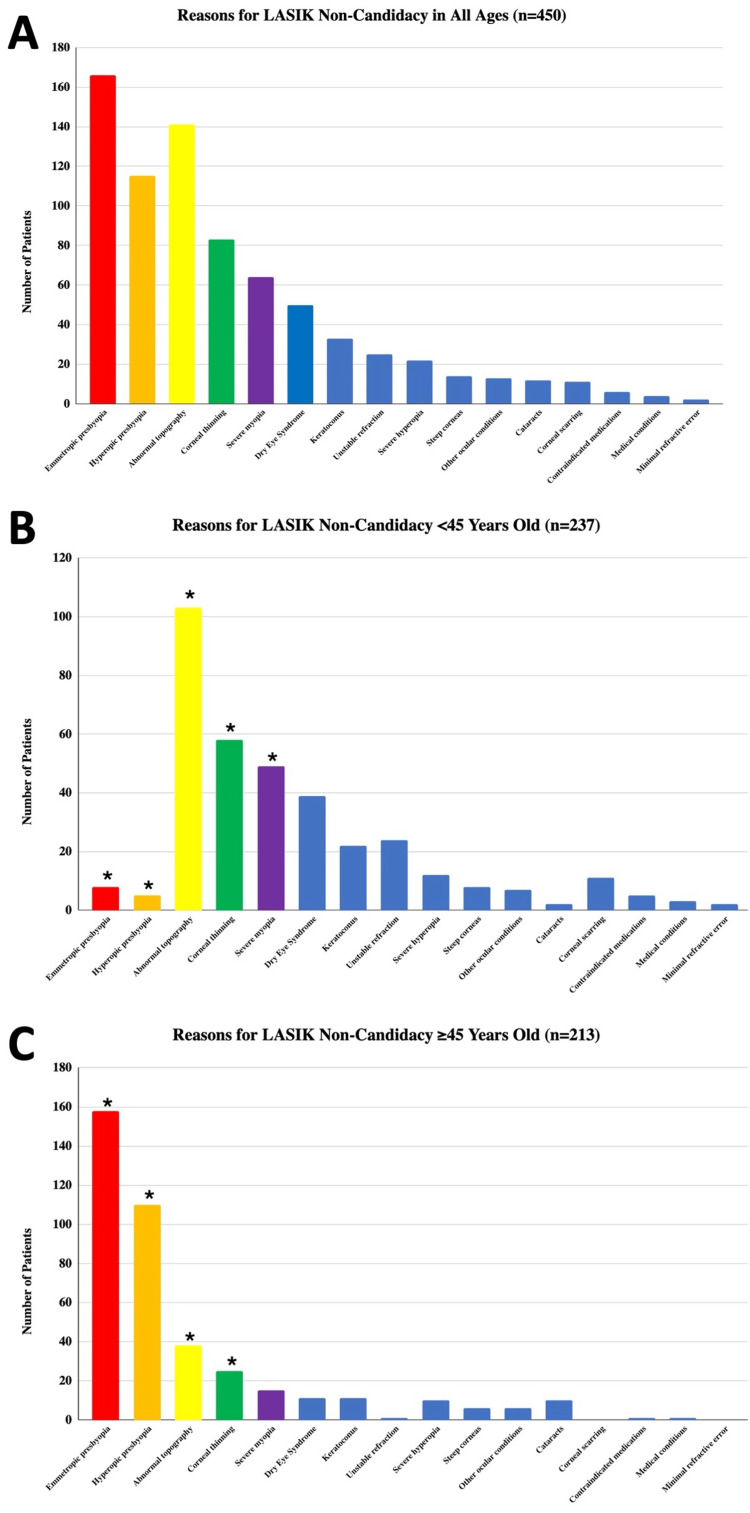
Reasons for Laser Assisted In Situ Keratomileusis (LASIK) Non-Candidacy in All Patients (A), Patients <45 (B), and Patients ≥45 Years Old (C) *Statistically significant difference between patients <45 and ≥45 years old using the chi-square test (P < 0.001).

For all patients, the top three reasons for LASIK non-candidacy were emmetropic presbyopia (166 patients, 36.9%), abnormal topography (141 patients, 31.3%), and hyperopic presbyopia (115 patients, 25.6%) (Figure 1A, Table 3). Out of the 450 patients who were LASIK non-candidates, 125 had a documented recommendation for an alternative surgery, including ICL, photorefractive keratectomy (PRK), RLE, or SMILE. From these 125 patients, 27 patients (21.6%) received ICL, 28 patients (22.4%) received PRK, 44 patients (35.2%) received RLE, and 26 patients (20.8%) underwent SMILE.

In the younger cohort (n = 237), the most common reasons for non-candidacy were abnormal topography (43.5%, n = 103), corneal thinning (24.5%, n = 58), and severe myopia (20.7%, n = 49) (Figure 1B). In contrast, among patients ≥ 45 (n = 213), emmetropic presbyopia (74.2%, n = 158) and hyperopic presbyopia (51.6%, n = 110) were the leading causes, followed by abnormal topography (17.8%, n = 38) (Figure 1C). Compared to older patients (≥ 45 years), those < 45 had significantly higher rates of abnormal topography (+25.7%), corneal thinning (+12.8%), and severe myopia (+13.7%), whereas emmetropic (+70.8%) and hyperopic (49.5%) presbyopia was significantly more frequent in patients ≥ 45 (all P < 0.001).

**Table 3:** Breakdown of LASIK Non-Candidacy by Subcategory for All Patients LASIK: laser assisted in situ keratomileusis.

Subcategory	Number of Patients (N)	Percentage out of 450 Patients
Emmetropic Presbyopia	166	36.9%
Hyperopic Presbyopia	115	25.6%
Abnormal Topography	141	31.3%
Corneal Thinning	83	18.4%
Severe Myopia	64	14.2%
Dry Eye Syndrome	50	11.1%
Keratoconus	33	7.3%
Unstable (Changing) Refraction	25	5.6%
Severe Hyperopia	22	4.9%
Steep Corneas	14	3.1%
Other Ocular Condition(s)	13	2.9%
Cataracts	12	2.7%
Corneal Scarring	11	2.4%
Contraindicated Medications	6	1.3%
Medical Condition	4	0.9%
Minimal Refractive Error	2	0.4%

## Discussion

LASIK is one of the most commonly used surgeries to correct myopia, astigmatism, and hyperopia [20,21]. Its popularity may be attributed to its efficacy, minimal discomfort, and recommendations from friends and family members [22]. Previous studies have been performed to characterize the primary reasons for rejection in different practices. We provide an updated characterization of LASIK non-candidacy reasons among all patients who expressed interest at a single-site refractive surgery center, to increase awareness that not all patients with refractive error are ideal candidates for LASIK.

Our study found an overall non-candidacy rate of 69.4% for LASIK, which is higher than prior studies, such as those by Bamashmus et al. and Alsulami et al., which demonstrated rejection rates of 25.4% and 24.9% in their Yemeni and Saudi cohorts, respectively [12,13]. Many potential reasons exist for this discrepancy, but it is important to note that several of the factors discussed in our study are not absolute exclusions for LASIK. Rather, they are relative and may vary based on the judgment of the refractive surgeon and their discussions with patients. The different demographics of this practice’s patient population may account for some of the discrepancy. In particular, the mean age of participants in our study was 41 years (range: 18-61), which is greater than the study conducted in the Yemeni population, with a mean age of 25 years (range: 16-51) [12]. This association can be further demonstrated by the difference in rejection rate based on age: patients under 45 had a rejection rate of 60%, while those 45 and older had a significantly higher rate of 84%. However, even within the younger subgroup in our study, patients aged 20-30 still had a 60% rejection rate. This suggests that age may not be the only factor contributing to the higher overall LASIK non-candidacy rate observed in our study.

The top three reasons for LASIK non-candidacy in our cohort of patients under 45 years were abnormal topography, corneal thinning, and severe myopia, findings supported by previous studies [12-15]. This cohort of younger patients had steeper and thinner corneas compared to LASIK candidates of the same age cohort, which can inherently increase the risk of developing keratectasia [23-28]. Conversely, among patients aged 45 and older, the leading causes for LASIK non-candidacy were emmetropic presbyopia, hyperopic presbyopia, and abnormal topography. This may be attributed to the age-related loss of accommodation and early lenticular changes, such as cataract formation, which are relative exclusions to LASIK [29].

While a strict exclusion criterion exists, interpretation may vary between practices and surgeons. In our study, individuals with myopia greater than -8.00 D were excluded from LASIK, whereas previous studies used cutoffs greater than -11.00 D [12]. Additionally, other studies excluded patients with a CCT less than 480 µm due to the inherently thinner corneas in the studied population, whereas our study excluded anyone with a CCT less than 500 µm [12,13]. Ongoing advancements in risk assessments and preoperative screenings have led to increasingly stringent safety criteria for LASIK candidacy. These include the introduction of the Randleman criteria, additional indices for anterior and posterior corneal curvature, epithelial mapping, percent tissue alteration, biomechanical indices, and a residual stromal bed cutoff of 300 μm, rather than 250 μm, all of which have contributed to reported lower candidacy rates [25,30-32]. Notably, previous studies that demonstrated lower rates of LASIK non-candidacy used different instruments for measuring CCT, such as ultrasonic pachymetry, while we used a newer instrument, the Scheimpflug Pentacam [12]. The advent of advanced imaging modalities has refined the ability to detect subclinical keratoconus, which appropriately excludes patients from undergoing LASIK. For instance, the Belin/Ambrósio enhanced ectasia display deviation, a Pentacam screening index, has achieved high diagnostic accuracy for keratoconus screening, with a sensitivity of 92.96% and specificity of 89.62% [33]. In our study, patients < 45 years experienced a lower non-candidacy rate, which may reflect the advancements in diagnostic imaging that allow earlier detection of subclinical keratoconus compared to older patients. In addition, the prevalence of keratoconus varies by region, which may have affected our results. A higher prevalence of keratoconus has been observed in our population, possibly related to geographic factors such as increased exposure to ultraviolet light, which leads to oxidative effects on the cornea [34]. These factors likely contributed to the observed difference in LASIK candidacy rates between our studies and those reported previously.

The limited longevity and tendency for regression associated with hyperopic LASIK are well-recognized in the literature. Compared to myopic LASIK, its outcomes have consistently fallen short by being less predictable, more transient, and exacerbated by presbyopia [35]. Many surgeons have attempted to address their frustration with presbyopia, particularly in emmetropic presbyopes, by exploring corneal inlays such as the Raindrop Near Vision Inlay (ReVision Optics, Lake Forest, CA) or the Kamra Inlay (AcuFocus, Inc., Irvine, CA). Despite entering the market with great enthusiasm, these implants fell short of expectations, resulting in their discontinuation after receiving FDA approval [36]. The limitations of hyperopic LASIK increase the likelihood that some patients will require additional refractive procedures, especially as accommodation declines with age. Furthermore, undergoing hyperopic LASIK may limit their eligibility for future surgical options, such as corneal refractive procedures or cataract surgery [37]. LASIK monovision correction is a viable option for appropriately chosen patients; however, it should be implemented cautiously to prevent non-candidacy and the potential need for enhancement procedures. Patients, therefore, require a thorough and stringent preoperative evaluation to determine the most appropriate procedure.

We acknowledge the possibility of surgical bias in our practice, as the surgeons may have a preference against hyperopic LASIK in middle-aged patients. This may have influenced the rate of patients not deemed candidates for LASIK. Surgeons in our practice may have opted to forgo LASIK in patients with low-level hyperopia and/or presbyopia and chose other alternatives, such as RLE, due to concerns about the predictability and longevity associated with hyperopic LASIK [38-41]. Additionally, patients with high myopia were often considered for alternative procedures such as an ICL, which are subject to different criteria used in corneal refractive screening [42]. Nevertheless, if we still consider our overall rate of non-candidacy for refractive surgery, rather than just for LASIK, the total number of non-candidates would be 325 patients instead of 450. This is because 125 patients underwent other refractive procedures such as PRK, SMILE, ICL, and RLE. This adjustment results in a lower overall non-candidacy rate for refractive surgery of 50.2% (325 of 648 patients), compared to 69.4% for LASIK specifically. Our findings show how the availability of alternative procedures to patients who do not qualify for LASIK has influenced the overall non-candidacy rate.

Limitations of this study include its retrospective design and the potential influence of media exposure on vision corrective procedures, which extends to a greater number of middle-aged and presbyopic patients beyond the typical LASIK candidate demographic. Advertisements may target different demographics included in our practice than those in other studies, which may explain the higher mean age in our population. Another limitation is that corneal warpage was not a distinct exclusion criterion in our study. Some patients with abnormal corneal topography may have corneal warpage. Although we followed the recommended 2-6 weeks contact lens discontinuation period, this may have been insufficient for topographic normalization and LASIK eligibility. If these patients had returned after a longer discontinuation period, it may have lowered our rejection rate. Additionally, presbyopic patients were never offered a monovision trial and were included in our LASIK non-candidacy cohort. A successful monovision trial could have made them eligible for LASIK, potentially contributing to our higher non-candidacy rates. Ultimately, our results reflect the practice patterns of surgeons at a single institution in Draper, UT, which may differ from those of other centers and thus limit the generalizability of this study. Despite these limitations, the objective of this analysis is not to enforce stricter standards of practice but to propose potential alternatives that may be more appropriate for patients who are not ideal candidates for LASIK. By providing a comprehensive and updated assessment of LASIK non-candidacy based on a large sample size, this study aims to contribute to the growing literature on this topic.

Among refractive surgeries, LASIK has undergone some of the most extensive evaluation with great scrutiny by the FDA [1-6]. Through this process, LASIK has shown significant improvements across multiple parameters, including visual acuity, contrast sensitivity, and patient-reported visual symptoms such as glare and halos [13]. Patient satisfaction remains high with LASIK, as it is rare for patients to express regret after the procedure [35]. Its approval for use among the U.S. Air Force supports its established safety and efficacy among all corneal refractive surgeries [43]. We speculate that the observed lower rates of LASIK non-candidacy are not due to its inferiority or inherent complications, which exist with any ocular procedure, but rather to the stricter criteria to meet candidacy and the availability of newer alternative procedures. Identifying the most common reasons for LASIK non-candidacy across different patient demographics may help surgeons improve their selection of patients who are most likely to benefit from LASIK.

## Conclusions

The authors explore the rates of LASIK non-candidacy and associated reasons to better identify suitable candidates for this procedure. The most common reason for LASIK denial in our population at a single surgical center in Draper, UT, was presbyopia, likely due to concerns about postoperative hyperopic drift and lack of interest in monovision LASIK. Following presbyopia, our findings were most similar to those identified in the Midwestern U.S. population study, which also excluded patients based on abnormal topography and insufficient corneal thickness. Although LASIK typically provides freedom from glasses, patients should not expect to maintain uncorrected 20/20 vision due to the onset of myopic regression and/or presbyopia. The high non-candidacy rate observed in our study reflects the implementation of rigorous screening criteria and the availability of alternative procedures, allowing for more tailored refractive solutions based on individual corneal parameters.
